# Neonatal and congenital malaria: a case series in malaria endemic eastern Uganda

**DOI:** 10.1186/s12936-018-2327-0

**Published:** 2018-04-20

**Authors:** Peter Olupot-Olupot, Emma I. E. Eregu, Ketty Naizuli, Julie Ikiror, Linda Acom, Kathy Burgoine

**Affiliations:** 1 Neonatal Unit, Mbale Regional Referral Hospital, P.O. Box 1966, Mbale, Uganda; 2 Mbale Clinical Research Institute, Mbale, Uganda; 3 Busitema University, Faculty of Health Sciences, Mbale Campus, Mbale, Uganda

**Keywords:** Congenital malaria, Neonatal malaria, Clinical features, Uganda

## Abstract

**Background:**

Congenital malaria is the direct infection of an infant with malaria parasites from their mother prior to or during birth. Neonatal malaria is due to an infective mosquito bite after birth. Neonatal and congenital malaria (NCM) are potentially life-threatening conditions that are believed to occur at relatively low rates in malaria endemic regions. However, recent reports suggest that the number of NCM cases is increasing, and its epidemiology remains poorly described. NCM can mimic other neonatal conditions and because it is thought to be rare, blood film examinations for malaria are not always routinely performed. Consequently, many cases of NCM are likely to be undiagnosed. A retrospective chart review for all neonates admitted with suspected sepsis between January and July 2017 was conducted and noted four cases of NCM since routine malaria testing was introduced as part of standard of care for suspected sepsis at Mbale Regional Referral Hospital Neonatology Unit. This description highlights the need to conduct routine malaria diagnostic testing for febrile neonates in malaria endemic areas, and supports the urgent need to undertake pharmacological studies on therapeutic agents in this population.

**Case presentation:**

Four cases (two congenital malaria cases and two neonatal malaria cases) are described after presenting for care at the Mbale Regional Referral Hospital Neonatal Unit (Mbale RRH-NNU). The maternal age was similar across the cases, but both neonatal malaria cases were born to primigravidae. At presentation three cases had fever and history of fever, but one was hypothermic (34.8 °C) and no history of fever. One case of congenital malaria had low birth weight, while the other was born to an HIV positive mother. Both cases of congenital malaria presented with poor feeding, in addition one of them had clinical jaundice. The neonatal malaria cases presented in the third week compared to the congenital malaria cases that presented within 48 h after birth. All of the cases of NCM were treated with intravenous artesunate. The admitting clinicians also instituted a course of antibiotics empirically to cover against possible bacterial co-infections. All four cases recovered and were discharged alive.

**Conclusion:**

At the Mbale RRH-NNU, the finding of cases of NCM was not expected, therefore, neonates presenting with features of suspected sepsis in malaria endemic settings should be routinely screened for NCM. There is currently a lack of appropriate guidelines for treatment of NCM in the era of artemisinin-based combination therapy (ACT), therefore, efforts to establish the safety profile and efficacy of ACT anti-malarials in neonates to guide development of evidence-based treatment guidelines for NCM are needed.

## Background

Uganda remains a malaria high burden country, with eastern Uganda experiencing perennial high malaria transmission with > 100 infective bites per person per year [1, 2]. In endemic areas where mothers have acquired considerable immunity to malaria, infection with *Plasmodium falciparum* during pregnancy does not always cause symptomatic illness [3]. Congenital malaria results from transplacental transmission of malaria parasites from the mother to the baby in utero or during delivery. Its diagnosis is based upon detection of asexual forms of malaria parasites on a blood smear of the peripheral blood of the newborn, or later if there is no possibility of postpartum infection through infective mosquito bites [4]. However, these definitions are not precise if time is not tagged to them to enable differentiate congenital from neonatal malaria. Furthermore, precision of definitions would be achieved by using temporal relationship with PCR testing for malaria in the mother and their neonate. The reported incidence of congenital malaria in endemic regions varies widely from 0 to 37% [5–7]. It is widely believed that the placenta acts as an effective barrier preventing transfer of malaria parasites. However, even in the absence of congenital malaria, placental malaria significantly increase the risk of perinatal morbidity and mortality including low birth weight, intrauterine growth restriction, preterm labour and intrauterine fetal death [8]. Malaria in pregnancy is estimated to account for 100,000 neonatal deaths annually [3]. Maternal malaria can be prevented during pregnancy with intermittent presumptive treatment of malaria in pregnancy (IPTp) using sulfadoxine–pyrimethamine, and can reduce neonatal mortality by up to 61% [9].

It is also possible that maternal immunity to malaria may confer protection to the fetus through transmission of immunoglobulin G antibodies (IgG) against malaria [10]. The presence of fetal haemoglobin (HbF) in the neonate also prevents high parasitaemia [11]. However as maternal IgG and HbF in infants diminish with age, the infant’s susceptibility to *P. falciparum* increases. It is possible that this passive immunity may delay the onset or modify severity of symptoms by up to 6 weeks after birth making it hard to differentiate between congenital and neonatal malaria [12]. To maximize the chances of early detection of congenital malaria, neonates born to mothers with malaria in the last 7 days before delivery should be investigated with a blood film for malaria parasites irrespective of the clinical picture and weekly thereafter for the first month.

In the eastern region of Uganda, despite perennial malaria transmission, NCM is a rarely reported condition presumably because of low index of suspicion among clinicians, and greater emphasis on the diagnosis and treatment of neonatal sepsis (NS). Although it is recommended that neonates be routinely tested if mother was known to suffer from malaria in the 7 days before delivery, in practice it is often not routine to test neonates for malaria and, therefore, many cases may be missed. In neonates, the historical and most common symptom of malaria is fever [13]. Other symptoms and signs differ from those in older children with malaria, the clinical features of neonatal and congenital malaria overlap with sepsis syndromes [14]. Other symptoms can include anaemia, jaundice, diarrhoea, vomiting, lethargy, convulsions, irritability, tachypnoea, respiratory distress, hepatosplenomegaly [14]. Clinical descriptions and outcomes of NCM remain poorly documented even in malaria endemic areas where descriptions in infants, older children and adults have over the times progressed. More comprehensive descriptions inclusive of NCM are needed, especially from areas with intense transmission, and serially over time.

At Mbale Regional Referral Hospital IPTp is routinely administered to pregnant mothers, however, not all pregnant mothers in the region served by this hospital attend antenatal care. At the hospital NNU there are estimated 200 admissions per month. During the study period June–December 2017 routine blood slides for malaria for all neonates admitted with fever had been introduced. This report is on four cases of NCM that highlight the presenting clinical features and their outcomes in a perennially malaria high transmission area in eastern Uganda. This has shown that malaria is a potentially missed diagnosis or co-morbidity in neonatal illnesses in malaria endemic areas.

## Case presentation

In this study congenital was differentiated from neonatal malaria based on the time of presentation from birth. Congenital malaria, as was previously defined, was used to classify cases 1 and 2 (Table 1). However, this description modified the definition for neonatal malaria that was applied by Runsewe-Abiodun et al. in Nigeria [15]. Therefore, for cases 3 and 4 (Table 1), this study considered symptoms attributable to malaria with evidence of ring forms of malaria parasite in the erythrocyte of an infant within the 8th–28th days of life. The maternal age was similar across all the 4 cases, range 24–28 years. The two neonatal malaria cases (3 and 4) were born to primigravidae who had no recent history of fever in the 14 days to delivery. Three of the cases presented with fever or history of fever, while one of the congenital cases was hypothermic (34.8 °C), possibly due to the concurrent prematurity. This same infant was also born to an HIV+ mother. Whereas there are many risk factors for preterm deliveries, it is possible that in this mother either the HIV [16], or antiretroviral drugs [17], may have contributed to both prematurity and susceptibility to malaria in this baby. The other congenital malaria case had clinical jaundice and pyogenic meningitis. The jaundice in this case 1 was attributed to a number of possible factors including neonatal jaundice, sepsis, malaria or a combination of these. Poor breastfeeding was noted in both congenital cases. All of the cases of NCM were treated with intravenous artesunate 4 mg/kg at 0, 12, 24 h then daily for 7 days. Since sepsis could not be excluded due to lack of laboratory investigative capacity, the admitting clinicians also instituted a course of broad-spectrum antibiotics empirically to cover against possible bacterial infections. All cases recovered and were discharged alive.

**Table 1:** Summary of admission characteristics and outcomes of neonatal and congenital malaria

Variable	Congenital malaria		Neonatal malaria	
	Case 1	Case 2	Case 3	Case 4
Mother				
Age (years)	28	24	24	26
HIV status	Negative	Positive	Negative	Negative
Parity	2 + 1	3 + 0	1 + 0	1 + 0
Fever within 14 days of delivery	Yes	Yes	No	No
Neonate				
Age (days)	2	1	14	16
Sex	M	F	M	F
Birth weight (kg)	2.7	1.7	3.5	2.8
Admission weight (kg)	2.4	1.7	3.9	2.5
Symptoms				
Fever	Yes	No	Yes	Yes
Duration of fever (days)	1	0	3	2
Fast breathing	Yes	No	No	Yes
Breastfeeding	No	No	Yes	Yes
Poor feeding	Yes	Yes	No	No
Signs				
Temperature (°C)	40.1	34.8	36.1	39.1
Jaundice	Yes	No	No	No
Pallor	Yes	No	No	No
Respiratory rate (breath/m)	104	Not taken	Not taken	72
Heart rate (BPM)	225	108	166	113
SP0_2_ (%)	96	79	96	68
Hepatomegaly	No	No	No	No
Splenomegaly	No	No	No	No
Tachycardia	Yes	No	Yes	No
Laboratory				
CSF				
Proteins (g/L)	0.3	No	No	< 0.1
Sugar (mmol/L)	8.12	No	No	10.08
WBC	75 × 10^6^/L	No	No	< 5 × 10^6^/L
Malaria				
Blood slide	Positive	Positive	Positive	Positive
Treatment				
Antibiotics^a^	Cefotaxime/Genta	Amp/Genta	Amp/Genta	Amp/Genta
Anti-malarial	Artesunate	Artesunate	Artesunate	Artesunate
Blood transfusion	No	No	No	No
Oxygen	No	Yes	No	Yes
Outcome				
At discharge	Alive	Alive	Alive	Alive

*Ampi/Genta* ampicillin and gentamycin, *BPM* beats per minute, *°C* degree celsius, *g/L* grams per litre, *kg* kilogram, *L* litre, *mmol* millimole, *WBC* white blood cell

^a^Empirical treatment

## Discussion and conclusions

In this case series, a description is made of both congenital and neonatal malaria in a setting that introduced routine neonatal malaria testing in a malaria endemic area. This case series shows that NCM malaria is more common than previously thought [18]. It is also a reminder that NCM still exists despite IPTp and other malaria preventive measures, and its diagnosis may be missed especially when malaria screening measures are not put in place in NNUs in malaria endemic areas. Other reports suggest that the incidence of NCM may be increasing. Proper descriptions of NCM are important to ensure more comprehensive understanding of the clinical spectrum and outcomes of malaria in neonates. Malaria endemicity has been suggested to play a role in the prevalence of NCM. There are reports suggesting in hyperendemic areas the prevalence of NCM is higher [5, 15], while others are contrary [7]. It is however, plausible epidemiologically that in settings of intense perennial transmission the mothers have developed herd immunity, their newborn babies have protective antibodies to the disease, and therefore the prevalence is negligible [10]. Some underlying factors may be responsible for the equipoise observed in this case series. For instance, primigravidae are known to have a high risk for malaria compared to multigravidae [19]. In addition, the role of maternal malaria in congenital malaria infections has been traced to infections in the third trimester [15]. It is also possible that co-morbidity that damages the placenta in utero may contribute to the risk of the congenital malaria. HIV co-infected pregnant women may have impaired antibody response and have been shown to have a significantly increased risk of placental malaria [20]. This is consistent with some reports suggesting that HIV increases the chances of vertical transmission of malaria [16, 20]. Maternal low age has been reported to influence congenital malaria [19]. In this series, all the mothers were within the same age range, but their parities differed.

Although rapid malaria tests have been used for the diagnosis of NCM, the gold standard for the diagnosis of NCM is the detection of parasites in the Giemsa-stained peripheral blood smear [21]. The Mbale RRH-NNU recently introduced routine testing for malaria using blood smears in neonates reporting with signs of sepsis. Consequently, all cases identified with NCM were managed with good outcomes.

On treatment, amodiaquine, chloroquine and sulfadoxine–pyrimethamine have all been successfully used in Nigeria to treat neonates with malaria [22]. Although artemisinin-based combination therapy (ACT) is the recommended treatment for uncomplicated malaria in infants, the neonates have been largely excluded from ACT clinical trials. There are, therefore, limited data available on the use of ACT in neonates and many of them carry label restrictions for neonates [23]. For infants weighing less than 5 kg with uncomplicated *P. falciparum*, the World Health Organization (WHO) recommends treatment with ACT at the same mg/kg body weight dose as for children weighing 5 kg. The WHO acknowledges too that most anti-malarials lack infant formulations, which can lead to either under or over dosing. In addition, infants can deteriorate rapidly therefore there should be a low threshold for parenteral treatment. Many anti-malarials are frequently used off-label based on the dosing schedule for older children [24], but have not reported evidence of serious toxicity [23].

Due to the physiological immaturity and rapid changes that occur in neonates, the pharmacokinetic and dynamic (PK/PD) profiles of anti-malarial drugs are likely to be different to older children. Slow gastric emptying, villous formation and intestinal motor activity, which do not mature until week 20 of life, affect the enteral absorption of most medications [25]. Parenteral treatment is preferable for neonates and young infants. Differentiating congenital from neonatal malaria based on temporal relationship to birth may be strengthened by inclusion of DNA PCR for both the mother and her newborn to determine the source of the infection. But in resource limited areas there are no routine DNA PRC testing services due to prohibitive capital, running and maintenance costs, except in research settings. The temporal characteristic would help interpret the role of physiological immaturity in neonates for future PK/PD studies on anti-malarial drugs in this age group, and how these determine treatment outcomes.

## Limitations

This is only a case series with case definitions based on temporal relationships from birth to case presentation. A larger study with capacity to conduct molecular, parasite count and PK/PD testing, and long term follow up would help better refine definitions, outcomes and interpretation of these findings. Nonetheless, this report has been able to demonstrate that NCM still exists. The definitions used have set pace in appropriate description of the spectrum of disease in this age group and will strengthen interpretation of anti-malarial PK/PD studies in this population in relation to the physiological immaturity.

## Conclusion

In summary, NCM is an important diagnosis to consider in any newborn with clinical features of NS to a mother in a malaria-endemic area. It is possible that in the absence of routine malaria testing many neonates are dying before malaria is diagnosed. In areas with malaria endemicity the burden of NCM may be underestimated. Malaria test should be incorporated as routine test in neonates with suspected sepsis so as not to miss NCM. Early and correct diagnosis of NCM is crucial as infants are at increased risk of rapid disease progression, severe malaria and death. Additional efforts are needed to establish the safety profile and efficacy of ACT in neonates to guide the development of evidence-based treatment guidelines for NCM. Furthermore, for pregnant mothers who test malaria positive in their late gestational period weekly malaria testing of their babies as follow up mechanism for surveillance of NCM should be done.

## References

[1] Katureebe A, Zinszer K, Arinaitwe E, Rek J, Kakande E, Charland K (2016). Measures of malaria burden after long-lasting insecticidal net distribution and indoor residual spraying at three sites in Uganda: a prospective observational study. PLoS Med..

[2] Kilama M, Smith DL, Hutchinson R, Kigozi R, Yeka A, Lavoy G (2014). Estimating the annual entomological inoculation rate for *Plasmodium falciparum* transmitted by *Anopheles gambiae* s.l. using three sampling methods in three sites in Uganda. Malar J..

[3] Eisele TP, Larsen DA, Walker N, Cibulskis RE, Yukich JO, Zikusooka CM (2012). Estimates of child deaths prevented from malaria prevention scale-up in Africa 2001–2010. Malar J..

[4] Menendez C, Mayor A (2007). Congenital malaria: the least known consequence of malaria in pregnancy. Semin Fetal Neonatal Med..

[5] Ekanem AD, Anah MU, Udo JJ (2008). The prevalence of congenital malaria among neonates with suspected sepsis in Calabar, Nigeria. Trop Doct..

[6] Lehner PJ, Andrews CJ (1988). Congenital malaria in Papua New Guinea. Trans R Soc Trop Med Hyg.

[7] Quinn TC, Jacobs RF, Mertz GJ, Hook EW, Locklsey RM (1982). Congenital malaria: a report of four cases and a review. J Pediatr.

[8] Osungbade KO, Oladunjoye OO (2012). Prevention of congenital transmission of malaria in sub-Saharan African countries: challenges and implications for health system strengthening. J Trop Med..

[9] Menendez C, Bardaji A, Sigauque B, Sanz S, Aponte JJ, Mabunda S (2010). Malaria prevention with IPTp during pregnancy reduces neonatal mortality. PLoS ONE.

[10] Riley EM, Wagner GE, Akanmori BD, Koram KA (2001). Do maternally acquired antibodies protect infants from malaria infection?. Parasite Immunol.

[11] Billig EM, McQueen PG, McKenzie FE (2012). Foetal haemoglobin and the dynamics of paediatric malaria. Malar J..

[12] Mohan K, Mr BJ, Singh RD, Maithani MM, Chaurais RN (2016). The clinico-hematological features and management outcome in neonatal malaria: a nine years analysis from North India. Curr Ped Rev..

[13] Covell G (1950). Congenital malaria. Trop Dis Bull..

[14] D’Alessandro U, Ubben D, Hamed K, Ceesay SJ, Okebe J, Taal M (2012). Malaria in infants aged less than six months—is it an area of unmet medical need?. Malar J..

[15] Runsewe-Abiodun IT, Ogunfowora OB, Fetuga BM (2006). Neonatal malaria in Nigeria—a 2 year review. BMC Pediatr..

[16] Zack RM, Golan J, Aboud S, Msamanga G, Spiegelman D, Fawzi W (2014). Risk factors for preterm birth among HIV-infected Tanzanian women: a prospective study. Obstet Gynecol Int.

[17] Powis KM, Kitch D, Ogwu A, Hughes MD, Lockman S, Leidner J (2011). Increased risk of preterm delivery among HIV-infected women randomized to protease versus nucleoside reverse transcriptase inhibitor-based HAART during pregnancy. J Infect Dis.

[18] Mukhtar M (2007). The growing incidence of neonatal malaria–a situational review in developing countries. Niger J Med..

[19] Kochar DK, Thanvi I, Joshi A, Subhakaran, Aseri S, Kumawat BL (1998). Falciparum malaria and pregnancy. Indian J Malariol.

[20] Perrault SD, Hajek J, Zhong K, Owino SO, Sichangi M, Smith G (2009). Human immunodeficiency virus co-infection increases placental parasite density and transplacental malaria transmission in Western Kenya. Am J Trop Med Hyg.

[21] Falade C, Mokuolu O, Okafor H, Orogade A, Falade A, Adedoyin O (2007). Epidemiology of congenital malaria in Nigeria: a multi-centre study. Trop Med Int Health..

[22] Hyacinth HI, Oguche S, Yilgwan CS (2012). Summary description of 24 cases of neonatal malaria seen at a tertiary health center in Nigeria. Iran J Pediatr..

[23] World Health Organization (2010). Guidelines for the treatment of malaria.

[24] Larru B, Molyneux E, Ter Kuile FO, Taylor T, Molyneux M, Terlouw DJ (2009). Malaria in infants below six months of age: retrospective surveillance of hospital admission records in Blantyre, Malawi. Malar J..

[25] Kearns GL, Abdel-Rahman SM, Alander SW, Blowey DL, Leeder JS, Kauffman RE (2003). Developmental pharmacology—drug disposition, action, and therapy in infants and children. N Engl J Med.

